
JOURNAL INFORMATION
==============================
NLM Title Abbreviation: ScientificWorldJournal
Journal ID: ScientificWorldJournal
NLM Journal ID: 101131163
Journal ID: TSWJ

The Scientific World Journal

ISSN: 2356-6140
EISSN: 1537-744X
Publisher: Wiley

ARTICLE INFORMATION
==============================
PMCID: PMC5956454
PMID: 15349555
DOI: 10.1100/tsw.2004.85
Article ID: 167412
Article version: 1
Subjects: Research Article

The Management of Nonseminomatous Testicular Cancer

Steele Graeme S.
Richie Jerome P.
Brigham and Women's Hospital and Harvard Medical School, USA
Academic Editor: Anthony Atala

Publication date: 2004
Electronic publication date: 2004 Jun 7
Volume: 4
First page: 316
Last page: 329
Copyright: Copyright © 2004 Graeme S. Steele and Jerome P. Richie.
Copyright year: 2004
License: This is an open access article distributed under the Creative Commons Attribution License, which permits unrestricted use, distribution, and reproduction in any medium, provided the original work is properly cited.
License URL: https://creativecommons.org/licenses/by/3.0/

==============================
Articles
*TheScientificWorldJOURNAL* (2004) 4 (S1), 316–329
ISSN 1537-744X; DOI 10.1100/tsw.2004.85

### The Management of Nonseminomatous Testicular Cancer

Graeme S. Steele, MBBCh, FCS and Jerome P. Richie, MD, FACS
*Brigham and Women's Hospital and Harvard Medical School*

**E-mails**: gsteel@partners.org

*Previously published in the Digital Urology Journal*

**DOMAIN**: urology

### INTRODUCTION

Despite the fact that testis cancer accounts for only 1% of all male malignancies, it is the most common solid malignancy affecting males between the ages of 15 to 35 years. The management of germ cell tumors (GCT) of the testis has proved to be a model of success among solid tumors. This is due to tremendous advances during the past 2 decades, which have changed the once dismal prognosis for patients with advanced disease.[1] Testicular cancer has, in fact, become one of the most curable of all solid neoplasms. Thirty years ago testicular cancer accounted for 11.4% of all cancer deaths in the 25 to 34 year age group, with an overall 5 year survival rate of 64%.[2] The most recent five-year survival rate for GCT of the testis in the United States was recently reported as being in excess of 90%.[3]

Improved prospects for cure and long-term disease free survival rate, are related to a better understanding of the natural history of testicular tumors, improved staging methods and surgical techniques, as well as to the introduction of effective platinum based combination chemotherapy.[4] Modifications to the surgical management of testis cancer have significantly reduced morbidity associated with the classical full bilateral retroperitoneal lymph node dissection. Template and nerve sparing retroperitoneal node dissections are now routinely employed in low stage nonseminomatous germ cell tumors (NSGCT), providing the patient with the benefit of accurate pathological staging combined with reduced morbidity. In addition, nerve sparing techniques have also been used in dissections for residual disease following chemotherapy.[5]

Chemotherapy for advanced GCT has undergone a number of important breakthroughs during the past three decades. In the 1960's the first major chemotherapeutic advance in metastatic NSGCT was described; a combination of chlorambucil, methotrexate and dactinomycin produced a response rate of 30%.[6] By the 1970's combination therapies such as vinblastine and bleomycin had been shown to produce objective responses; 57% complete response rates and 45% long-term disease-free survival rates were reported.[7]

The discovery of cisplatin as a highly effective anti-neoplastic agent in 1965,[8] and its later use in germ cell tumors of the testis (GCT), was another important breakthrough. In 1974 the combination of cisplatin, vinblastine and bleomycin was investigated by the Indiana group.[9] Of the 47 patients in the initial phase II study, 100% achieved a response rate. Thirty five (74%) patients achieved complete remission, the remaining patients achieved a partial remission.

In 1983 the combination of bleomycin, etoposide, and cisplatin (BEP) was first described by a group of investigators at the Royal Marsden Hospital.[10] This combination was later compared in randomized studies to cisplatin, vinblastine and bleomycin (PVB) and found to be both less toxic and more effective. The use of BEP therefore became a milestone in the treatment of GCT of the testis and has become a widely used treatment for advanced disease.[11-13]

Since the late 1980's investigators have distinguished between low (good) risk and high (poor) risk disease, based on the diverse prognosis of patients with advanced GCT.[14-15] In those patients with good risk disease, the objective is to reduce treatment toxicity while maintaining high rates of complete response. In this regard three cycles of BEP were found to be as effective as four cycles of BEP, in patients with good risk disease.[14] On the other hand, studies in poor risk patients have focused on improving both response rates and survival. Numerous phase II and phase III studies have not led to very significant improvements in outcome in poor risk patients, who are fortunately rarely encountered today.[16-17]

### PRINCIPLES OF SURGICAL MANAGEMENT OF TESTIS CANCER

The principles which underlie the modern surgical treatment of GCT are based on the fact that testis cancer spreads in a predictable and stepwise fashion, with the notable exception of choriocarcinoma. The spermatic cord contains four to eight lymphatic channels that ascend into the retroperitoneum and fan out medially into the retroperitoneal lymph node chain. The first echelon of lymph nodes draining the right testis is located within the interaortocaval nodes at the level of the second vertebral body. The first echelon of nodes draining the left testis is located in the para-aortic region in an area bounded by the renal vein superiorly, aorta medially, ureter laterally and the origin of the inferior mesenteric artery inferiorly. Following spread to either the left or right primary echelon of nodes, subsequent spread may occur in a retrograde fashion to the common, external and inguinal nodes, or cephalad via the cysterna chyli, thoracic duct and supraclavicular nodes. Despite the predictability of lymph node metastasis in testis cancer, there is a failure rate of 5% with distant metastasis following node negative retroperitoneal lymphadenectomy (RPL).[18] This is probably due to the fact that testicular lymphatics may very occasionally bypass retroperitoneal lymph nodes altogether and communicate directly with the thoracic duct. In a review of 104 consecutive cases of stage II NSGCT, Donohue et al, made a number of important observations confirming the predictability of lymphatic spread in testicular cancer.[19] He reported that suprahilar lymph node spread was rare in stage IIA disease, unlike stage IIB disease, where suprahilar spread was not uncommon. In low stage disease the absence of contralateral nodal involvement was noted. In addition, in this series gonadal vein involvement was noted in a significant number of cases in both low and high stage II disease. These observations have important implications for the surgical management of testis cancer.

Lymphatics of the epididymis drain into the external iliac chain; therefore, in locally extensive disease, epididymal involvement may be associated with positive pelvic nodes. Testis cancer that involves the scrotum may result in inguinal node metastases; in addition, prior scrotal surgery or retrograde spread from extensive retroperitoneal involvement may also cause inguinal metastases. Scrotal violation in the setting of orchiectomy for testis cancer has generally been condemned as compromising patient prognosis and for this reason, inguinal orchiectomy with high ligation of the spermatic cord has been the standard of care in the initial management of testis cancer for almost 100 years.[20] However in a recent review regarding the implications of scrotal violation in testis cancer, Capeluto et al reported on a meta-analysis of 1,182 patients, scrotal violation had occurred in 206 patients. They found no statistical differences in distant recurrence or survival, implying that scrotal violation may not necessarily carry a worse prognosis.

Distant spread of testis cancer occurs most commonly to the pulmonary region, with intraparenchymal pulmonary involvement. Subsequent spread is to the liver, viscera, brain and bone, with bony secondaries being encountered late in the course of the disease.[21]

In summary, with the notable exception of choriocarcinoma, testicular cancer generally spreads in a predictable pattern. This has led to the development of new surgical techniques, which provide accurate pathological staging while at the same time providing therapeutic benefit. These techniques are also associated with reduced morbidity when compared to the classic full retroperitoneal lymphadenectomy (RPL).

### PREDICTION OF METASTATIC POTENTIAL

In general, patients with clinical stage I NSGCT have a 20 - 30% incidence of micrometasteses in retroperitoneal lymph nodes. In addition follow-up studies of these patients have shown that visceral metastases occur in approximately 10%. Therefore, in theory approximately 70% of patients with clinical stage I NSGCT could be cured by radical orchiectomy alone. Studies to investigate this dilemma were initiated in the United Kingdom in 1979, and in Denmark in 1980. [22,23] These studies showed that the relapse rate after orchiectomy alone was approximately 30%. More than 90% of relapses occur within the first 2 years after orchiectomy; median time to relapse was 4 to 5 months after orchiectomy. Less than 10% of relapses occur later than 2 years after orchiectomy. Fifty percent of relapses occur in the retroperitoneum and 30% of relapses occur without elevation of tumor markers. One third of relapses occur in the lung and 12% of relapses occur with elevation of tumor markers as the only sign of tumor recurrence.

Several studies have investigated prognostic factors that predict relapse. A study performed by the MRC in the United Kingdom described four prognostic factors;[24] vascular invasion, lymphatic invasion, the presence of embryonal carcinoma and the absence of yolk sac tumor. A prognostic index was subsequently created based on these factors. Patients with four positive prognostic factors were found to have a 46% risk of relapse compared with 21% for those with 2 factors and 16% for those with one factor. No relapses occurred in patients without any risk factors.[25] In addition, primary tumor extent has also been shown to have prognostic significance.[4] Clinical stage I NSGCT is therefore no longer managed as a homogeneous disease, rather treatment may now be individualized according to the likelihood of positive retroperitoneal lymph nodes. Patients with low risk of having positive nodes have the option of entering an observation protocol and thus may be spared the morbidity of adjuvant therapy. However, despite prognostic criteria, it is still not possible to establish highly accurate distinctions between patients who should and those who should not receive adjuvant therapy. In the MRC study in the United Kingdom the high risk group constituted less than 25% of all patients and most importantly less than half of all relapses.[23] In a recent report, the long term outcome results of a prospective surveillance trial for clinical stage I NSGCT were described. With a median follow-up of 11.3 years, 74.3% of patients remained disease free, while the remainder experienced relapse. All relapses occurred within 2 years, the median time to relapse was 5 months. Significant indicators of relapse during surveillance were a predominant embryonal carcinoma histology and vascular invasion. In patients with neither of these two risk factors, the relapse rate was 12%, none of these patients died of their disease.[26]

### STAGING TESTIS CANCER

There are both clinical and pathological staging systems for NSGCT (Tables 1 and 2). Most systems in use today are a variation on the one proposed by Boden and Gibb in 1951.[27] Low stage disease refers to clinical stages I (A) and IIA (B1) disease, whereas high stage disease encompasses stages IIB (B2), IIC (B3), and III (C) disease. This distinction between low and high stage disease is important, as it determines, to a large degree, the adjuvant therapy that the patient receives. While the clinical staging system is based predominantly on CT scan and chest X- Ray, pathologic staging is based on the surgical findings and the surgical specimens obtained at radical orchiectomy and RPL. The staging error associated with clinical staging is therefore removed by pathologic staging.

**TABLE 1**
**CLINICAL STAGING SYSTEMS FOR TESTICULAR CANCER**

| BODEN/GIBB: | ROYAL MARSDEN HOSPITAL: |
|---|---|
| A (I): Tumor confined to the testis | I |
| B (II): Spread to regional nodes | IIA: < 2cm<br>IIB: > 2cm, < 5cm<br>IIIC: > 5cm |
| C (III): Spread beyond retroperitoneal nodes | III: Supraclavicular, nodal or mediastinal involvement<br>IV: Extralymphatic involvement |

**TABLE 2**
**PATHOLOGICAL STAGING**

| American Joint Committee on Cancer TNM Classification | SKINNER |
|---|---|
| Primary tumor (T) - extent of the primary tumor is classified after radical orchiectomy<br>pTX - Primary tumor cannot be evaluated i.e. radical orchiectomy not yet performed.<br>pT0 - No evidence of primary tumor (histologic scar in the testis)<br>pTIS - Carcinoma in situ, i.e. pre-invasive stage<br>pT1 - Tumor limited to the testis, including the rete testis<br>pT2 - Tumor invades beyond the tunica albuginea or into the epididymis<br>pT3 - Tumor invades the spermatic cord<br>pT4 - Tumor invades the scrotum | A: Confined to the testis |
| Regional lymph nodes (N)<br>NX - Regional lymph nodes cannot be assessed<br>N0 - No regional lymph node metastasis<br>N1 - Metastasis in a single lymph node<br>N2 - Metastasis in a single lymph node 2 cm or 2cm, no extra-nodal extension less in greatest dimension, or multiple lymph nodes, none more than 5 cm in greatest dimension<br>N3 - Metastasis in a lymph node more than 5 cm in greatest dimension | B: Spread to the retroperitoneum<br>BI: < 6 positive nodes, no node ><br>BII: > 6 positive nodes, any node > 2 cm<br>BIII: massive retroperitoneal disease |
| Distant metastasis (M)<br>MX - Presence of distant metastasis cannot be assessed<br>M0 - No distant metastasis<br>M1 - Distant metastasis | C: Metastatic disease |

### RETROPERITONEAL LYMPHADENECTOMY (RPL) IN GCT OF THE TESTIS

### RPL-I

This dissection is performed on patients with no clinical signs of spread to the retroperitoneum, and no surgically visible disease, i.e. clinical stage I disease. The surgical technique employed is either a template or nerve sparing technique. Therefore sympathetic nerves responsible for antegrade ejaculation are preserved, resulting in normal ejaculatory function in the vast majority of patients undergoing RPL-I.

### RPL-II

This procedure is performed on patients with low volume clinically demonstrable disease, or visible disease at the time of surgery, i.e. clinical stage IIA or IIB disease. The surgical boundaries are generally wider than in RPL-I, usually bilateral above the inferior mesenteric artery and in most cases bilateral below the mesenteric artery as well. The lumbar sympathetics and the hypogastric plexus are carefully preserved, resulting in preservation of ejaculation in experienced hands in over 95% of patients undergoing RPL-II.

### RPL-III

This procedure is performed on patients who have failed primary therapy, either primary chemotherapy, or primary retroperitoneal node dissection and who have either retroperitoneal recurrences or a persistent retroperitoneal mass detected radiographically (usually by CT scan). In selected cases nerve sparing techniques maybe employed.[5]

### MANAGEMENT OF CLINICAL STAGE I DISEASE

- SURVEILLANCE VS ADJUVANT THERAPY

The decision to place a patient on a surveillance protocol or to opt for adjuvant therapy in the form of either RPL-I or chemotherapy depends on a number of factors, such as risk factors for disease relapse, patient compliance and local expertise with respect to performance of the surgical procedure.

- ADVANTAGES OF RPL-I

1. Thirty percent of patients with clinical stage I NSGCT have pathologic stage II disease.
  2. Accurate pathologic staging allows for earlier definition of further treatment options.
  3. RPL-I is both a staging and a therapeutic procedure.
  4. Follow-up after RPL-I is significantly less intensive than patients followed on surveillance protocols and therefore less expensive.
  5. Anxieties, which inevitably accompany the uncertainties associated with a surveillance protocol, are eliminated by surgical intervention.

### MANAGEMENT OF CLINICAL STAGE II DISEASE

The most appropriate therapy for patients with clinical stage II NSGCT is controversial. Debate centers around the role of RPL-II vs chemotherapy and also around the role of post RPL chemotherapy.

There is general agreement that clinical stages IIC and III disease be managed initially with chemotherapy, and that surgery should be reserved for residual retroperitoneal masses. The initial management of clinical stages IIA and IIB disease, however, remains controversial. At the Brigham and Womenís Hospital most patients who present with clinical stage IIA disease undergo RPL-II. Furthermore, patients with < 3cm of nodal involvement also undergo RPL-II, while chemotherapy is generally recommended for those patients with > 3cm of nodal involvement.

- ADJUVANT CHEMOTHERAPY FOLLOWING RPL-II

The role of adjuvant chemotherapy following RPL-II vs. an expectant approach has been addressed by a number of investigators.[28,29] Richie reviewed 39 patients with pathologic stage II disease who were carefully followed post RPL-II. Relapses occurred in only 8% of patients, all of whom were rendered disease free with 3 to 4 cycles of chemotherapy.[28] In this study therefore immediate post RPL-II chemotherapy would have benefited only a small number of patients. In contrast, a randomized multi-center study reported a 48% relapse rate for patients with pathologic stage II disease, following RPL-II.[30] These two studies highlight the fact that results from institutions where RPL is performed frequently, may not parallel results from large cooperative trials in which some centers contribute relatively small numbers of patients.

Following RPL-II the decision to administer two cycles of chemotherapy depends on a number of factors: tumor volume, number of positive nodes, presence of vascular invasion in the primary tumor and histology of the primary tumor. Expectant management is appropriate in patients with fewer than 6 positive nodes (all of which are less than 2cm in diameter), with no positive margins. For patients with more than 6 positive nodes or high volume disease, two cycles of chemotherapy are given following RPL-II.[31] This protocol has achieved excellent remission rates. For patients who develop a recurrence while being treated expectantly, chemotherapy is employed at that stage, once again with excellent remission rates.

### SURGICAL MANAGEMENT OF ADVANCED NSGCT: RPL-III

RPL-III in patients with advanced NSGCT provides essential information for the future management of the patient:

- Evaluates the response to chemotherapy.
- Removes viable GCT, thus achieving a complete response to chemotherapy and surgery.
- Directs the need for further therapy.

Approximately 30% of patients with high volume metastatic NSGCT will experience a partial remission following cisplatin based chemotherapy and are therefore candidates for RPL-III, in addition to those patients who recur in the retroperitoneum following an incomplete node dissection for low stage disease. Partial remission is defined as normal tumor markers in combination with radiographic evidence of disease in the chest, abdomen, mediastinum, neck or elsewhere.

### Persistent or recurrent retroperitoneal mass: Histopathology and implications.

Early retrospective studies revealed that RPL-III defines three subsets of patients based on histopathological analysis of the resected specimen: 40% - necrosis/fibrosis; 40% - adult teratoma; 20% - residual NSGCT.[32,33] Therefore approximately 60% of patients with evidence of a residual mass on post-chemotherapy imaging studies will either have viable cancer or teratoma. A more recent study showed that the likelihood of malignancy in postchemotherapy resected tumor was 13%, with the remainder of tumor specimens containing teratoma or necrosis.[34]

The finding of only necrotic/fibrotic tissue implies that no further treatment is required.[35] However patients with viable GCT require an additional two cycles of chemotherapy. Fox et al[36] reported a series of 27 patients who received additional cisplatin-based chemotherapy whereas 7 did not. Disease free survival was 70% in the chemotherapy group, while all 7 patients who did not receive additional chemotherapy relapsed. The rationale to resect residual teratoma is multi-factorial: firstly, by resecting residual teratoma the growing teratoma syndrome is avoided, in which indolent growth causes complications from local progression;[37] secondly, malignant transformation of mature teratoma to sarcoma and adenocarcinoma which is resistant to chemotherapy has been well described;[38] thirdly, there is a risk of recurrent viable GCT in teratoma, with no difference in incidence between mature and immature teratoma.[39]

### RPL-III: Indications and Patient Selection

Despite the well described hazards of residual viable GCT in the retroperitoneum following chemotherapy, as well as recurrent retroperitoneal GCT following RPL-I or RPL-II, the indications for RPL-III are controversial and there are no clear guidelines. The presence of elevated tumor markers, however, remains the only generally accepted contraindication to adjunctive surgery in patients after chemotherapy.[40] The management of patients whose tumor markers have normalized following chemotherapy ranges from observation, irrespective of the results of imaging studies, to surgery for all patients.[41,42] A residual mass is usually detected by CT scan following chemotherapy; however, the definitions of a normal CT scan differ from one institution to another. Definitions include: no visible mass, lymph nodes no larger than 1 cm and lymph nodes less than 1.5 cm to 2 cm in diameter.[37]

In view of the well described complications related to RPL-III, patient selection plays an important role in excluding those patients who are unlikely to have viable disease. Donohue et al[43] found that in cases where no teratomatous elements were detected in the primary tumor and a greater than 90% decrease was seen in the volume of retroperitoneal disease by sequential CT scans, viable germ cell tumor or teratoma was not seen in the resected specimen. Fossa et al[42] reported on 37 patients who underwent RPL-III despite negative CT scans following chemotherapy; teratoma was found in 30% while 3% were found to have NSGCT. The volume of teratoma was small, and may thus not be clinically significant to justify a blanket policy of RPL-III despite negative CT scan findings. Toner et al[34] reported on 185 patients who underwent RPL-III after chemotherapy; in this series, no factors could be found that could reliably predict patients who had residual viable malignancy or teratoma in the retroperitoneum. In view of this RPL-III was recommended for all patients with residual abnormalities on imaging of the retroperitoneum, and in all patients with an initial mass of greater than 3 cm regardless of the post chemotherapy radiological findings.[32,33] An international data set was recently collected comprising data from six study groups. Logistic regression analysis was used to estimate the probability of necrosis and the ratio of cancer and mature teratoma, in patients who were treated with cisplatin-based chemotherapy for metastatic NSGCT, and who obtained normal tumor markers prior to resection. Multivariate combination of predictors yielded reliable models, which discriminated necrosis well from other histologies, but which discriminated cancer only reasonably well from mature teratoma.[44]

While there are statistical correlationís with various factors such as degree of tumor shrinkage, size of residual mass, pre-chemotherapy tumor markers and teratomatous elements in the primary specimen, the risk of a false negative prediction is approximately 20%.[34,42]

### Complications of RPL III

RPL-III is a technically very demanding procedure, which sometimes entails a combined abdominal and thoracic approach. Loss of ejaculation has been well documented in patients undergoing RPL-III. In a recent study 25.9% of patients were reported to have absence of ejaculation following RPL-III. In this study, the volume of tumor resected was higher in those patients who had absence of ejaculation than in those patients with preservation of ejaculation. In addition, absence of ejaculation was reported significantly more often in patients with right paracaval/interaortocaval tumor (34.5%), than in those with left para-aortic tumor (16.7%).[45]

Nerve sparing techniques have been successfully employed in a subset of these patients.[5] In a recent review of 472 patients who underwent RPL-III, 93 (19.7%) were subjected to nerve sparing procedures, mean size of residual disease on CT scan prior to the procedure was 6.3 cm (range 1 to 19). In this subset of patients 76.5% reported normal ejaculation and ten pregnancies were conceived. Testis cancer recurred in 6 patients but no tumor was retroperitoneal. Therefore, nerve sparing RPL-III appears to be viable in a subset of patients with limited volume and lateralized disease, without compromising cancer surgery outcomes, while preserving ejaculation.[5]

Occasionally, resection of the infrarenal vena cava is necessary and has been well described.[46,47] In addition, aortic tube grafts may be necessary to replace aortic segments which are damaged by subadvential dissection.[48] The complication rate is therefore directly related to the complexity of the dissection; in addition, complications related to the cumulative toxicity of chemotherapeutic agents are of particular relevance to patients undergoing RPL-III following chemotherapy.

Of particular relevance to post-chemotherapy patients is the association of restrictive lung disease in patients who have received bleomycin as part of their chemotherapy protocol. Bleomycin induced pulmonary toxicity is associated with a mortality rate which approaches 50%. In a recent report, Baniel et. al.[49] reviewed the surgical morbidity of 603 patients who underwent RPL after primary chemotherapy for stage II and III testis cancer. The complication rate was 20.7% and the mortality rate was 0.8%. Pulmonary complications were the most frequent cause of severe morbidity: 6 patients developed adult respiratory distress syndrome, the underlying cause of which was a combination of bleomycin induced pulmonary toxicity, together with large volume retroperitoneal and pulmonary disease resected in this subset of patients.

### CHEMOTHERAPY FOR PATIENTS WITH ADVANCED NSGCT

While chemotherapeutic trials were being conducted, it became clear that prognoses varied and that patients should therefore be divided into low and high risk categories. For those patients predicted to have a more favorable outcome i.e. good risk, the goals have been to maintain high cure rates while reducing treatment related toxicity.[3,50] On the other hand the goal of treating poor risk patients has been to improve the proportion of patients achieving a complete response, while at the same time achieving tolerable treatment side effects. Using data from the cisplatin, vinblastine, bleomycin (PVB) studies, the Indiana University staging system was developed (Table 3). This classification uses clinical parameters to divide patients with disseminated testis cancer into three stages - minimal, moderate and advanced disease. While minimal and moderate disease is good risk and is associated with high complete response rates to cisplatin based chemotherapy and low risk of relapse, advanced disease is poor risk disease and therefore has a lower complete response rate and a higher rate of relapse. Although the Indiana University system is easy to use it does not take into account the important role that the tumor markers HCG (human chorionic gonadotropin), AFP (alpha fetoprotein) and LDH (lactate dehydrogenase) play in this disease.[50] Bosl et al developed one of the first validated models to mathematically categorize patients into prognostic groups.[51] The variables in the model include the number of metastatic sites as well as HCG and LDH. Several other models have been published affirming the principles that the Bosl model described.[50,52] The International Germ Cell Cancer Collaborative Group recently published a clinically based prognostic classification for GCT in which the following adverse factors were identified: mediastinal primary site; degree of elevation of alpha-fetoprotein (AFP), human chorionic gonadtrophin (hCG) and lactate dehydrogenase (LDH); presence of nonpulmonary metasteses such as liver, bone and brain. Integration of these factors produced the following groupings: good prognosis, comprising 60% of GCT with a 91% 5-

**TABLE 3**
**INDIANA UNIVERSITY STAGING SYSTEM FOR DISSEMINATED DISEASE: MINIMAL AND MODERATE STAGES CONSTITUTE GOOD RISK DISEASE**

| |
|---|
| Minimal |
| 1. Elevated markers only |
| 2. Cervical nodes (± nonpalpable retroperitoneal nodes) |
| 3. Unresectable, nonpalpable retroperitoneal disease |
| 4. Fewer than 5 pulmonary metastases per lung field and largest < 2 cm (± nonpalpable retroperitoneal nodes) |
| Moderate |
| 5. Palpable abdominal disease mass only |
| 6. Moderate pulmonary metastases: > 5 - 10 metastases per lung field and largest < 3 cm or solitary pulmonary metastasis of any size > 2 cm (± nonpalpable retroperitoneal nodes) |
| Advanced |
| 7. Advanced pulmonary metastases: > 10 pulmonary metastases per lung field, or multiple pulmonary metastases with largest > 3 cm (± nonpalpable retroperitoneal nodes) |
| 8. Palpable abdominal mass plus supradiaphragmatic disease |
| 9. Liver, bone, or CNS metastasis |

year survival rate; intermediate prognosis, comprising 26% of GCT with a 79% 5-year survival rate; and poor prognosis, comprising 14% of GCT with a 48% 5-year survival rate[53] (Table 4).

Hopefully the widespread use of a standardized prognostic model will result in more patients benefiting from chemotherapeutic regimens with fewer side effects.[53,54]

### CHEMOTHERAPY IN GOOD RISK PATIENTS

The first prospective trials in good risk patients compared the VAB-6 regimen (vinblastine, dactinomycin, bleomycin, cyclophosphamide and cisplatin) with a two drug regimen of etoposide plus cisplatin (EP).[55] The results of this trial demonstrated equivalence but with a toxicity favoring EP. In long term follow-up no excess of late relapse was found in patients treated with 4 cycles of EP.[14] Thus 4 cycles of EP with cycles repeated every 21 - 28 days has become one of the international standards for good risk disease.

Einhorn et al compared 4 cycles of BEP (bleomycin, etoposide, cisplatin) to 3 cycles of BEP. One hundred eighty-four patients were entered into this trial. Overall 99% of patients with minimal advanced stage disease and 95% of patients with moderate advanced stage disease achieved disease free status. Only 5.6% of patients relapsed and the overall disease free survival was the same in both arms of this study, which demonstrated that elimination of the fourth cycle of BEP reduced toxicity without affecting survival.[14]

In a trial conducted by the Eastern Cooperative Oncology Group (ECOG), 178 good risk patients were randomized to receive three cycles of EP either with or without bleomycin.[56] Myelosuppression was slightly greater in the BEP arm (57% versus 45%); no pulmonary toxicity was seen in either group. This trial was stopped early because of a statistically significant increase in the number of adverse events (defined as death, persistent or progressive cancer, or relapse) in patients not receiving bleomycin. Of the 86 patients treated with BEP, 95% achieved a disease-free status and 86% were continuously disease free, compared with 90% and 70% respectively in the EP arm. This data supports the necessity of including bleomycin when three cycles of cisplatin based chemotherapy are used.

**TABLE 4**
**INTERNATIONAL GERM CELL CONSENSUS CLASSIFICATION FOR NSGCT:**

| | |
|---|---|
| GOOD PROGNOSIS: | Primary tumor - testis or retroperitoneal<br>and<br>No non-pulmonary visceral metastases<br>and<br>Good markers - all of:<br>AFP < 1000ng/ml and<br>hCG < 5000 iu/l and<br>LDH < 1.5 x upper limit of normal |
| INTERMEDIATE PROGNOSIS: | Primary tumor - testis or retroperitoneal<br>and<br>No non-pulmonary visceral metastases<br>and<br>Intermediate markers - any of:<br>AFP > 1000 and < 10,000 ng/ml or<br>hCG > 5000 and < 50,000 iu/l or<br>LDH > 1.5 x N and < 10 x N |
| POOR PROGNOSIS: | Mediastinal Primary<br>or<br>Non-pulmonary visceral metastases<br>or<br>Poor markers - any of:<br>AFP > 10,000 ng/ml or<br>hCG > 50,000 iu/l or<br>LDH > 10 x upper, limit of normal |

Carboplatin was chosen as a substitute for cisplatin in a trial performed by Bajorin et al., because of its better toxicity profile, i.e. fewer renal, otologic and neurologic toxicities.[57] A durable complete response was achieved in only 76% of patients treated with etoposide and carboplatin (EC) compared to a durable complete response rate of 87% in patients treated with EP.[35] This trial therefore confirmed the efficacy of four cycles of EP and also showed that carboplatin cannot be substituted for cisplatin in this subset of patients.

### CHEMOTHERAPY IN POOR RISK PATIENTS

Clinical trials involving patients with advanced disease have focused on enhancing response rates and improving survival. Poor risk patients are fortunately rare and usually present with primary mediastinal GCT.[58] Marked elevation of one or more tumor markers (beta-human chorionic gonadotropin, alpha-fetoprotein and lactate dehydrogenase) is a common reason for inclusion into the poor risk group. In addition the presence of liver, bone or brain metastases as well as increasing bulk of disease are also used as inclusion criteria for the poor prognostic group.[58,59] Although there are a large number of phase II studies for the treatment of poor risk patients, none of them has been sufficiently encouraging to be considered as a standard therapy.

Several trials have been conducted using conventional dose chemotherapy. Ifosfamide in combination with etoposide and cisplatin (VIP) has been compared to BEP.[60] VIP proved to be more toxic but equivalent to BEP with respect to efficacy.

Impressive results have been reported from single center studies describing the use of cyclic alternating chemotherapy regimens. These regimens expose the GCT to a wide variety of chemotherapeutic drugs in the hope of preventing drug resistance. The results however have not been validated by randomized trials.

Due to the fact that high dose chemotherapy given as second or third line therapy was associated with a very high morbidity and mortality rate, the role of chemotherapy with autologous bone marrow transplantation (ABMT) was investigated.[61,62] Twenty eight poor risk patients were entered into a trial in which high dose chemotherapy was given to those patients whose tumor markers exhibited a prolonged decline following standard dose therapy. When the results in this subset of patients were compared to the prior experience in poor risk patients, there appeared to be a survival benefit.[63] A French cooperative group reported their experience with high dose chemotherapy followed by ABMT. Patients were randomized to receive either conventional or high dose chemotherapy in combination with ABMT. However, 28% of patients randomized to receive high-dosed therapy did not receive this treatment and no statistical differences were apparent between the 2 groups of patients.[17]

Growth factor and peripheral blood stem cell support have been used in combination with dose intensification of all cycles of chemotherapy. In a study involving 77 poor risk patients, dose escalation of the chemotherapy regimen was combined with granulocyte-macrophage-colony stimulating factor (GM-CSF). Overall 67% of patients achieved progression-free survival.[64]

### CHEMOTHERAPY FOR RELAPSED TESTIS CANCER

For the 20% to 30% of patients with advanced GCT, who do not achieve complete response to first line chemotherapy, second line salvage chemotherapy remains an option.[65,66]

The current standard chemotherapy that serves as a basis for comparison is vinblastine, ifosfamide and cisplatin (VeIP).[66] As second line therapy 33% to 69% of patients achieved a complete response with ifosfamide based regimens, which was found to be durable in approximately 50% of patients.[67] In view of this, ifosfamide is generally considered to be a standard first line salvage therapy. Approximately 25% to 50% of patients who relapse following chemotherapy have been shown to achieve a complete response to high dose carboplatin and etoposide with and without oxazophosphorine (cyclophosphamide, ifosfamide) and ABMT.[68] Forty three percent of patients achieved a complete response, 27% remained alive and disease free at 8 months of follow-up.

Favorable pretreatment prognostic factors for survival to treatment with conventional dose salvage therapy include a prior complete response to cisplatin based chemotherapy and a testicular primary site. Prognostic factors that predict an inferior long term disease free survival include an initial incomplete response to cisplatin based chemotherapy and a primary mediastinal site of disease.[68] These poor prognostic factors predict a very poor outcome and the role of standard chemotherapy is thus limited. Aggressive surgery may offer a reasonable alternative as may radiotherapy, in addition high dose chemotherapeutic regimens may have a role.[35]

### CONCLUSIONS

Advances in the management of GCT of the testis have led to a multimodal approach which has resulted in the vast majority of patients being cured of their disease. More than 90% of patients with newly diagnosed GCT will be cured. RPL not only provides accurate data for pathological staging, but is also therapeutic in low stage disease. Moreover RPL plays an essential role in the management of high stage disease with respect to both directing further patient management and providing long-term disease free survival following complete resection of residual teratoma. Precise surgical technique however is mandatory to ensure disease free survival. Patients presenting with advanced disease are stratified into good, intermediate and poor risk groups. Therapy for good risk disease consists of either four cycles of EP or three cycles of BEP; resulting complete response rates are high. On the other hand poor risk patients require intensive and specialized management and should be referred to an appropriate center with expertise in this area. ABMT or stem cell support combined with high dose chemotherapeutic regimens (VeIP) can be successfully used to treat patients who relapse following first line chemotherapy. However patients who relapse with poor prognostic features do not respond well to high dose chemotherapy, and may thus require aggressive surgery or radiotherapy.

### REFERENCES

1. Einhorn, L. H.: Treatment of testicular cancer: a new and improved model. J. Clin. Oncol., 8: 1777, 1990.
2. Oliver, R.T.D: Clues from the natural history and results of treatment supporting the monoclonal origin of germ cell tumors. Cancer Surv., 9:333, 1990.
3. Bosl ,G.J., Motzer, R.J: Testicular germ cell cancer. N Engl J Med., 337: 242, 1997.
4. Levin, M.: Prognostic features of primary and secondary germ cell tumors. Urol. Clin .N. Amer., 20: 39, 1993.
5. Coogan, C.L., Mohamed, J.H., Wahle, G.R., Foster, R.S., Rowland, R.G., Bihrle, R, Donohue, J.P: Nerve sparing post-chemotherapy retroperitoneal lymph node dissection for advanced testicular cancer. J Urol., 156: 1656, 1996.
6. Li, M., Whitmore, W., Golbey, R., Grabstaldt, H.: Effects of combined drug therapy on metastatic cancer of the testis. JAMA., 174: 1291, 1960.
7. Samuels, M. L., Johnson, D. E. and Holoye, P. Y.: Continuous intravenous bleomycin (NSC-125066) therapy with vinblastine (NSC-49842) in stage III testicular neoplasia. Cancer Chemother. Rep., part 1, 59: 563, 1975.
8. Rosenberg, B., Van Camp, L., Krigas, T.: Inhibition of cell division in E. Coli by electrolysis products from a platinum electrode. Nature 205: 698, 1965.
9. Einhorn, L. H and Donohue, J.: Cis-diaminedichloroplatinum, vinblastine and bleomycin combination chemotherapy in disseminated testicular cancer. Ann Intern Med., 87: 293, 1977.
10. Willaims, S. D., Birch, R., Einhorn, L. H., Irwin, L., Greco, F. A., Loehrer, P., J.: Treatment of disseminated germ cell tumors with cisplatin, bleomycin and either vinblastine or etoposide. N. Engl. J. Med., 316: 1435, 1987.
11. Peckham,M., Barrett, A., Liew, K., Horwich, A., Robinson, B., Dobbs, H.J., McElwain, T.J., Hendry, W.F.: The treatment of metastatic germ-cell testicular tumors with beleomycin, etoposide and cisplatin (BEP). Br J Cancer., 47:613-619, 1983.
12. Williams, S.D., Birch, R., Einhorn, L.H, Irwin. L., Greco, F.A., Loehrer, P.J.: Treatment of disseminated germ cell tumors with cisplatin, bleomycin and either vinblastine or etoposide. N Engl J Med., 316: 1435, 1987.
13. Bajorin, D.F., Geller, N.L., Weisen, S.F., Bosl, G.J. : Two drug therapy in patients with metastatic germ cell tumors. Cancer., 67: 28, 1991.
14. Einhorn, L.H., Williams, S.D., Loehrer, P.J., Birch, R., Drasga, R., Omura, G., Greco, F.A.: Evaluation of optimal duration of chemotherapy in favourable-prognosis disseminated germ cell tumors: a southeastern cancer group protocol. J Clin Oncol., 7: 387, 1989.
15. Stoter, G., Sleijfer, D.T.H., Schornagel, J.H., Bokkel-Huinink., W.W., Vermeiljen, K., Sylvester, R.: BEP versus VIP in intermediate risk patients with disseminated nonseminomatous testicular cancer (NSTC). Proc Am Soc Clin Oncol., 12: 714, 1993.
16. Nichols, C.R., Williams, S.D., Loehrer, P.J., Greco, A., Crawford, E.D., Weetlaufer, J., Miller, M.E., Bertolucci, A., Schacter, L., Einhorn, L.H: Randomised study of cisplatin dose intensity in poor risk germ cell tumors: a Southeastern cancer study group and southwest oncology group protocol. J Clin Oncol., 9: 1163, 1993.
17. Chevreau, C., Droz, J.P., Pico, J.L., Biron, P., Kerbrat, P., Heron, J.F., Fargeot, P., Bouzy, J.: Early intensified chemotherapy with autologous bone marrow transplantation in first line treatment of poor risk nonseminomatous germ cell tumors. Eur. Urol., 23: 213, 1993.
18. Whitemore, W.F. Jr: Germinal Tumors of the testis: In proceedings of the Swedish National Cancer Conference. Philadelphia J.B. Lipincott Co. pp 485, 1973.
19. Donohue, J.P, Zachary, J.M., Maynard, B.R: Distribution of nodal metastases in nonseminomatous testis cancer. J Urol., 128: 315, 1982.
20. Keber, G.M.: Sarcoma of the testicle, conclusion based upon 114 cases. Amer J. Med., 117: 535, 1899.
21. Johnson, D.E., Appele, C., Samuels, M.L., Luna, M.: Metastasis from testicular carcinoma. Urology 8: 234, 1976.
22. Rorth, M., Jacobsen, G.K., Maase H von der.: Surveillance alone versus radiotherapy after orchiectomyfor clinical stage I nonseminomatous testicular cancer. J Clin Oncol., 9: 1543, 1991.
23. Read, G., Stenning, S.P., Cullen, M.H: Medical Research Council prospective study of surveillance for stage I testicular cancer. J Clin Oncol., 10: 1761-1768, 1992.

24. Peckham, M.J., Barret, A., Horwich, A., Hendry, W.F: Orchiectomy alone for stage I testicular nonseminoma. Br J Urol., 55: 754, 1983.
25. Freedman, L.S., Parkinson, M.C., Jones WG: Histopathology in the prediction of relapse in patients with stage I testicular teratoma treated by orchidectomy alone. Lancet 2: 294, 1987.
26. Sogani, P.C., Perrotti, M., Herr, H., Fair, W.R., Thaler, H.T., Bosl, G: Clinical stage I testis cancer: Long term outcome of patients on surveillance. J Urol., 159: 885, 1998.
27. Boden, G., Gibb, R: Radiotherapy and testicular neoplasms. Cancer., 2: 294-297, 1951.
28. Richie, J.P., Kantoff, P.W.: Is adjuvant chemotherapy necessary for patients with stage BI testicular cancer. J Clin Oncol., 9(8): 1393-1398, 1991.
29. Pizzocaro, G., Monfardin, N.: Adjuvant chemotherapy in selected patients with pathologic stage II NSGCT. J Urol., 131:667, 1983.
30. Williams, S.D., Stablain, D.M., Einhorn, L.M.: Immediate adjuvant chemotherapy versus observation with treatment at relapse in pathological stage II testicular cancer. N Engl J Med 317:1433, 1987.
31. Weisbach, L., Mamhapp, J.M.: Adjuvant chemotherapy for metastatic stage NSGCT of the testis. J Urol., 146:1295, 1991.
32. Brenner, J., Vugrin, D., Whitmore, W.F: Cytoreductive surgery for advanced nonseminomatous germ cell tumors of the testis. Urology., 19: 571 - 575, 1982.
33. Einhorn, L.H., Williams, S.D., Mandelbaum, I., Donohue, J.P.: Surgical resection in disseminated testicular cancer following cytoreduction. Cancer., 48: 904 - 908, 1981.
34. Toner, G., Panicek, D.M., Heelan, R.T., Geller, N.L., Shiow-Yun, L., Bajorin, D.F., Motzer, R.J., Scher, H.I., Morse, M.J., Fair, W.R., Sogani, P.C., Whitemore, W.F., McCormack, P.M., Bains, M.S., Martini, N., Bosl, GJ: Adjunctive surgery after chemotherapy for nonseminomatous germa cell tumors: recommendations for patient selection. J Clin Oncol., 8: 1638 - 1694, 1990.
35. Law, T.M., Motzer, R.J., Bajorin, D.F., Bosl, G,J: The management of patients with advanced germ cell tumors.Urol Clin North Amer., 24: 773, 1994.
36. Fox, E.P., Weathers, T., Williams, S.D., Loehrer, P.J., Ulbright, T.M., Donohue, J.P., Einhorn, L.H: Outcome analysis for patients with persistent nonteratomatous germ cell tumor in postchemotherapy retroperitoneal lymph node dissections. J Clin Oncol., 11: 1294, 1993.
37. Logothesis, C.J., Samuels, M.L., Trindade, A., Johnson, D.E.: The growing teratoma syndrome. Cancer., 50: 1629, 1982.
38. Loehrer, P.J., Mui, S., Majdu, S.I: Teratoma following cisplatin based chemotherapy for nonseminomatous germ cell tumors. A clinicopathologic correlation. J Urol., 135: 1189, 1986.
39. Herr, H.W., LaQuaglia, M.P.: Management of teratoma. Urol Clin of North Amer., 20: 145, 1993.
40. Bajorin, D.F., Herr, H.W., Motzer, R.J., Bosl, G.J.: Current perspectives on the role of adjunctive surgery in combined modality treatment of patient with germ cell tumors. Semin Oncol., 19: 145: 158, 1992.
41. Levitt, M.D., Reynolds, P.M., Steiner, M.: Nonseminomatous germ cell testicular tumors: residual masses after chemotherapy. Br J Surg. 72: 1902, 1985.
42. Fossa, S.D., Ous, S., Lien, H.H., Steinwig, A.E.: Post-chemotherapy lymph node histology in radiologically normal patients with metastatic nonseminomatous testicular cancer. J Urol., 141: 557 - 559, 1989.
43. Donohue, J.P., Foster, R.S.: Management of retroperitoneal recurrences: seminoma and nonseminoma. Urol Clin North Amer., 21: 4; 761 - 772, 1993.
44. Steyerberg, E.W., Keizer, H.J., Fossa, S.D.: Prediction of residual retroperitoneal mass histology after chemotherapy for metastatic NSGCT: multivariate analysis of individual patient data from six study groups. J Clin Oncol., 13: 1177, 1995.
45. Gels, M.E., Nijboer, A.P., Hoekstra, H.J.: Complications of post-chemotherapy resection of retroperitoneal residual tumor mass in patients with NSGCT. Brit J Urol., 79: 263, 1997.
46. Donohue, J.P., Thornhill, J.A., Foster, R.S., Rowland, R.G,, Bihrle, R.: Resection of the inferior vena cava or intraluminal vena caval tumor thrombectomyduring retroperitoneal lymph node dissectionfor metatstatic germ cell cancer: indications and results. J Urol., 146: 346, 1991.
47. Donohue, J.P., Thornhill, J.A., Foster, R.S., Bihrle, R.: Vascular considerations in post chemotherapy retroperitoneal lymph node dissection: part I - vena cava. World J Urol., 12: 182 - 186, 1994.
48. Donohue, J.P., Thornhill, J.A., Foster, R.S., Bihrle, R.: Vascular considerations in post chemotherapy retroperitoneal lymph node dissection: part II. World J Urol., 12: 187, 1994.
49. Baniel, J., Foster, R.S., Rowland, R.S., Bihrle, R., Donohue, J.P.: Complications of post-chemotherapy retroperitoneal lymph node dissection. J Urol., 153: 976, 1995.
50. Bajorin, D., Katz, A., Chan, E., Geller, N.: Comparison of criteria for assigning germ cell tumor patients to good risk and poor risk studies. J Clin Oncol., 6: 786, 1988.
51. Bosl, G.J., Geller, N.L., Cirrincione, C., Vogelzang, N.J., Bosl, G.J.: Multivariate analysis of prognostic variables in patients with metastatic testicualr cancer. Cancer Res., 43: 3403, 1983.
52. Medical research council working party on testicular tumors. Prognostic factors in advanced nonseminomatous germ cell tumors: results of a multi centre study. Lancet., 1: 8, 1985.

53. International Germ Cell Consensus Classification: A prognostic factor-based staging system for metastatic germ cell cancers. International germ cell cancer collaborative group. J Clin Oncol., 15:2; 549, 1997.
54. Mead, G.M., Stenning, S.P.: Prognostic factors for metastatic germ cell cancers treated with platinum basec chemotherapy: International germ cell cancer collaborative group to standardize risk criteria. Proc Am Soc Clin Oncol., 13: 790, 1994.
55. Bosl, G.J., Geller, N.L., Bajorin, D., Leitner, S.P., Yogada, A., Golbey, R.B., Scher, H., Vogelzang, N., Auman, J., Carey, R., Fair, W.R., Herr, H., Morse, M., Sogani, P., Whitemore, W.: A randomized trial of etoposide + cisplatin versus vinblastine + bleomycin + cisplatin + cyclophosphamide + dactinomycin in patients with good prognosis germ cell tumors. J Clin Oncol., 6: 1231, 1988.
56. Loehrer, P.J., Elson, P., Johnson D.H: A randomized trial of cisplatin plus etoposide with or without bleomycin in favorable prognosis disseminated germ cell tumors: an ECOG study. Proc AM Soc Clin Oncol., 10: 169, 1991.
57. Bajorin., D.F., Sarosdy, M.F., Pfister, D.G., Mazumdar, M., Motzer, R.J., Scher, H.I., Geller, N.L., Fair, W.R., Henry, H., Sagoni, P., Sheinfeld, J., Russo, P., Vlamis, V., Carey, R., Vogelzang, N., Crawford, E.D., Bosl, G.J.: Randomised trial of etoposide and cisplatin versus etoposide and carboplatin in patients with good risk germ cell tumors: A multiinstitutional study. J Clin Oncol., 11: 598, 1993.
58. Droz, J.P., Kramar, A., Rey, A.: Prognostic factors in metastatic disease. Semin Oncol., 19: 181, 1992.
59. Mead, G.M., Stemming, S.P.: Prognostic factors for metastatic germ cell cancers treated with platinum-based chemotherapy: the International Germ Cell Cancer Collaborative Group (IGCCCG) project to standardize risk criteria. Proc Am Soc Clin Oncol., 13: 790, 1994.
60. Loehrer, P.J,, Einhorn, L.H., Elson, P: Phase three study of cisplatin (P) plus etoposide (VP-16) with either bleomycin (B) or ifosfamide (I) in advanced stage germ cell tumors (GCT): An intergroup trial. Proc Am Soc Clin Oncol., 12: 831, 1993.
61. Nichols, C.R., Tricot, G., Williams, S.D., van Besin, K., Loehrer, P.J., Roth, B.J., Ackard, L., Hoffman, R., Gouler, R., Wolff, S.N.: Dose intensive chemotherapy in refractory germ cell cancer: A phase I/II trial of high dose carboplatin and etoposide with autologous bone marrow transplantation. J Clin Oncol., 7: 932, 1989.
62. Motzer, R.J., Mazumdar, M., Gulati, S.C., Gulati, S., Bajorin, D.F., Lyn, P., Vlamis, V., Bosl, GJ.: Phase II trial of high dose carboplatin and etoposide with autologous bone marrow transplantation in the first-line therapy for patients with poor-risk germ cell tumors. J Natl Cancer Inst., 85: 1828, 1993.
63. Rowland, R.G., Donohue, J.P.: Scrotum and testis. In: Gillenwater JY, Grayhack JT,Howards SS, Duckett JW eds. Adult and pediatric urology, 2nd ed. Chicago: Mosby-year book, 1565, 1991.
64. Bokemeyer, C., Schmoll, H.J., Harstrick. A., Illigen, H.J., Metzner, B., Roth, U., Hohnloser, J., Clemm, C., Berbel, W., Siegert, W., Ruther, U., Ostermann, H., Kneba, M., Hartlopp, J.H., Schroder, M., Poliwoda, H.: A phase I/II study of a stepwise dose-escalated regimen of cisplatin, etoposide and ifosfamide plus granulocyte -macrophage colony stimulating factor (GM - CSF) in patients with advanced germ cell tumors. Eur J Cancer., 16: 2225, 1993.
65. Einhorn, L.: Treatment of testicular cancer: a new and improved model. J Clin Oncol., 8: 1777, 1990.
66. Einhorn, L., Weathers, T., Loehrer, P.: Second line chemotherapy with vinblastine, ifosfamide and cisplatin after initial chemotherapy with cisplatin , VP-16, and bleomycin, (PVB-16B) in disseminated germ cell tumors (GCT). Proc Am Soc Clin Oncol ., 11: 196, 1992.
67. Harstrick, A., Schmoll, H.J., Wilke, H., Kohne, C.H., Stahl, M., Schober, C., Casper, J., Bruderek, L., Schmoll, E., Boekemyer. C,, Bergman. L., Lammers, U., Freund, M., Poliwoda, H.: Cisplatin, etoposide and ifosfamide salvage therapy for refractory or relapsing germ cell carcinoma. J Clin Oncol., 9: 1549, 1991.
68. Motzer, R.J., Bosl, G.J.: High dose chemotherapy for resistant germ cell tumors. Recent advances and future directions. J Natl. Cancer Inst., 84: 1703, 1992.

**This article should be referenced as follows:**

Steele, G.S. and Richie, J.P. (2004) The management of nonseminomatous testicular cancer. *TheScientificWorldJOURNAL* **4 (S1)**, 316–329.

**Handling Editor:**

Anthony Atala, Principle Editor for *Urology* — a domain of *TheScientificWorldJOURNAL.*
